# Whole-transcriptome analyses of Sorghum leaves identify key mRNAs and ncRNAs associated with GA_3_-mediated alleviation of salt stress

**DOI:** 10.3389/fpls.2022.1071657

**Published:** 2022-12-01

**Authors:** Yanqing Wu, Jiao Liu, Guisheng Zhou

**Affiliations:** ^1^ Joint International Research Laboratory of Agriculture and Agri-Product Safety, The Ministry of Education of China, Institutes of Agricultural Science and Technology Development, Yangzhou University, Yangzhou, Jiangsu, China; ^2^ Jiangsu Provincial Key Laboratory of Crop Genetics and Physiology, Yangzhou University, Yangzhou, Jiangsu, China

**Keywords:** sorghum, gibberellic acid (GA3), whole-transcriptome, salt stress, ceRNA

## Abstract

Sorghum has recently attracted much attention for its tolerance in high salt environment. However, the effect and regulatory mechanism of the gibberellic acid (GA_3_)-mediated alleviation of salt stress in sorghum remains unclear. Herein, we reported that a GA_3_ concentration of 50 mg/L is optimal for sorghum (“Jitian 3”) development under salt stress. We conducted a whole-transcriptome analysis between GA_3_-treated and control sorghum leaves under salt stress, and we identified 1002 differentially expressed (DE)-messenger RNAs (mRNAs), 81 DE-long non-coding RNAs (lncRNAs), 7 DE-circular RNAs (circRNAs), and 26 DE-microRNA (miRNAs) in sorghum following GA_3_ treatment. We also identified a majority of DE-mRNAs and non-coding RNAs (ncRNAs) targets that serve essential roles in phenylpropanoid biosynthesis and plant hormone networks. In addition, we generated a competitive endogenous RNA (ceRNA)-miRNA-target gene network, and 3 circRNAs (circRNA_2746, circRNA_6515, circRNA_5622), 4 lncRNAs (XR_002450182.1, XR_002452422.1, XR_002448510.1, XR_002448296.1) and 4 genes (LOC8056546, LOC8062245, LOC8061469, LOC8071960) probably act as valuable candidates for the regulation of the GA_3_-mediated alleviation of salt stress in sorghum. Our findings uncovered potential mRNA and non-coding RNAs that contribute to GA_3_ regulation, thus offering a basis for the future investigation of underlying mechanisms of salt stress in sorghum.

## Introduction

Salinity is a resource-based ecological challenge that is widely distributed, and is a major abiotic stressor affecting crop production everywhere (Song et al., 2011). In salt stress, crop roots cannot efficiently absorb water and nutrients from the soil, which damages plant cells, organs, and tissues, reduces metabolism, and promotes growth inhibition, which, in turn, reducing crop yield and quality (Negrao et al., 2017). Sorghum is an important source for food, brewing, energy, and forage production. It is one of the cereal crops with medium salt-tolerance capacity, and it is of great value to developing and utilizing saline soils. However, sorghum still encounters low emergence and suppressed metabolism in soils with high salt concentrations. Among the economic and efficacious approaches to improving and developing these saline soils is the screening of salt tolerant oil crops (Li et al., 2010). Studies have reported that the effect of salt stress on plant growth can be effectively mitigated to a specific range by the exogenous administration of hormonal gibberellins (Muniandi et al., 2018; Camara et al., 2018). Earlier studies demonstrated that gibberellic acid enhances seed germination, seed salt tolerance and minimizes the salt-mediated inhibition of seedling growth (Muhammad and Eui Shik, 2007; Nasri et al., 2012; Chunthaburee et al., 2014). It was also demonstrated that GA_3_ administration minimizes the harmful influences of salinity while increasing salinity resistance in plants (Ahmad, 2010). Sorghum (“Jitian 3”) is a salt-tolerant seed cultivar that has been commonly employed in China for over 40-50 centuries in arid, semiarid and water-logged areas. Although the regulatory mechanism behind sorghum response to salt stress is well-reported (Sun et al., 2020; Punia et al., 2021; Wu et al., 2022), the significance of gibberellins in sorghum development and underlying mechanisms under salt stress remains poorly understood.

High-throughput omics tools, such as metabolomics, proteomics and transcriptomics, are critical to determining the transcriptional modulation and metabolic parameters of salinity tolerance. Various plant salt-resistant genes were identified using transcriptome sequencing (Zhang et al., 2013; Zhang et al., 2020; Hussain et al., 2021; Wu et al., 2022). In several early transcriptome analyses of *Arabidopsis thaliana*, salt stress produced hundreds of differentially expressed genes (DEGs) (Zhang et al., 2013). Zhang et al. (2020) indicated that the *Cynanchum auriculatum* leaves have a relatively abundant gene expression and regulatory activities that cope with salt stress. Hussain et al. (2021) provided a detailed understanding of how TF pathways and ABA interact to cause stress responses is essential to improve tolerance to drought and salinity stress. Wu et al. (2022) identified important genes related to membrane lipid regulation in sweet sorghum under salt stress by transcriptome analysis. With the application of high-throughput sequencing technology, an increasing number of non-coding RNAs (ncRNAs) including long non-coding RNAs (lncRNAs), circular RNAs (circRNAs) and microRNA (miRNAs), have also been discovered in plant tissues. Such ncRNAs are present in *Arabidopsis*, *wheat*, *maize* and *rice*, and are known to serve critical functions in multiple biological processes involved in plant growth and stress response (Yu et al., 2019). However, no comprehensive studies have as yet characterized the transcriptome-wide distribution of ncRNA and mRNA in the sorghum, underscoring a novel direction for the ongoing molecular studies on the bottleneck of alleviating salt stress. Herein, our study determined the optimal GA_3_ concentration required for salt stress alleviation in sorghum (“Jitian 3”). Additionally, we performed a comparative whole-transcriptome analysis of sorghum leaves and identified the differentially expressed ncRNAs (circRNA, lncRNA, miRNA) and mRNAs related to GA_3_-mediated alleviation of salt stress. Based on these results and the prediction of the target transcript, we established a competitive endogenous RNA (ceRNA) network to indicate key mRNA–miRNA–lncRNA/circRNA interactions. Our findings will enhance our understanding of the role of exogenous gibberellic acid (GA_3_) in regulating salt tolerance in sorghum.

## Material and methods

### Plant materials, growth criteria and GA_3_ exposure

The homogeneous and healthy sorghum seed cultivar was “Jitian 3”, which was gifted to us by the Agricultural Research Institute in Hebei Province. Our research design followed a factorial format, with 1 salinity concentration (150 mM NaCl) and 5 GA_3_ concentrations (namely, 0, 25, 50, 75, and 100 mg/L). All experiments were completed three times. Healthy and plump seeds of the same size were disinfected with 1% sodium hypochlorite solution for 15 min, repeatedly rinsed with distilled water, and then placed in a glass petri dish with a cap of 15 cm in inner diameter and a filter paper of 15 cm in diameter. 100 seeds were evenly placed into the dish, and 50 ml of 1/2 Hoagland nutrient solution was injected. Each treatment was repeated 3 times. 10 ml of 150 mm NaCl treatment solution was added to each petri dish, and then the dishes were placed in an incubator with a photoperiod of 12h/12h (day/night) and a temperature of 25°C to promote germination, until the seedlings developed 1 leaf and 1 bud. Next, we planted seeds with similar bud lengths in a plastic tray (50 cm long; 30 cm wide; 5 cm high) with pre-formed air holes on the bottom. Then, all pots (5 cm in top diameter and 2.5 cm in bottom diameter) were filled with quartz sand, and up to 3 seeds were planted per pot approximately 3 cm deep, and 10 ml of GA_3_ solution of different concentrations was sprayed every 10 days on average. Data and samples were arbitrarily collected on day 40 from 1 of 3 replicates per group. Finally, these samples, along with 0 mg/L (control group) and 50 mg/L GA_3_ (treated group), were examined and used for further whole-transcriptome analyses.

### Morpho-physiological assessments

Plant samples were obtained on day 40 after planting. In total, 6 sorghum leaves were harvested from GA_3_-treated (GL_A, n= 3) and control plants (GL_B, n= 3) before rinsing with distilled water. We next measured the true leaf dimensions, as well as the leaf length and leaf width. After cleaning, the roots were further rinsed with distilled water before separating the plants into roots, stems, and leaves. The fresh weights were recorded. Dry weights were assessed after the samples were dried in an oven at 70 °C for 72 h until the plant achieved a constant weight for biomass assessment (Dai et al., 2012).

### Anatomical observation

The anatomical observation was performed as reported in the Zhao et al. (2020) publication. Fresh leaves were carved into 1 × 1 cm pieces before being fixed in 2.5% glutaraldehyde at 4°C for at least 4 h, followed by thrice rinsing in 0.1 M phosphate buffer for 15 min each, then another fixing in 1% osmium tetroxide for 4 h, with subsequent dehydration in 100% acetone and acetone that included anhydrous sodium sulfate for 15 min each, prior to embedding in Spurr resin. The specimens were carved again before double-staining with uranyl acetate and lead citrate. The mesophyll cells and chloroplasts were visualized and photographed using a transmission electron microscopy (TEM) (HT7700, HITACHI, Japan).

### RNA isolation and qualification

RNA isolation and qualification of Sorghum leaves were performed according to the previous reports (Sui et al., 2015). Total RNA isolation was done with TRIzol (Thermo Fisher Scientific, MA, USA), as per kit directions. RNA quality evaluations were performed on 1% agarose gels, whereas, RNA purity assessment employed a NanoPhotometer spectrophotometer (IMPLEN, CA, USA). RNA quantification was done with a Qubit RNA Assay Kit in a Qubit 2.0 Fluorometer (Life Technologies, CA, USA), and integrity assessment *via* the Agilent 2100 Bioanalyzer (Agilent Technologies, Santa Clara, CA, USA).

### RNA sequencing, DE-RNAs screening and DE analyses

All sequencing programs were performed by the OE Biotech Co., Ltd (Shanghai, China) (Cao et al., 2022). The circRNA-, lncRNA-, miRNA-, and mRNA-seq details are summarized in a prior publication (Shi et al., 2021). The raw data are in the NCBI Sequence Read Archive (SRA) database, under accession numbers PRJNA878791 and PRJNA858876.

### GO enrichment and network analysis

GO analysis was done *via* Blast2GO and the GO database (Conesa et al., 2005). KEGG network analysis was conducted using the KEGG network database (www.kegg.jp/kegg/kegg1.html) (Kanehisa and Goto, 2000).

### ceRNA axis generation

To elucidate the association among the mRNAs, miRNAs, lncRNAs, and circRNAs, a ceRNA modulatory axis was generated employing the circRNA/lncRNA−miRNA−mRNA data and the ceRNA hypothesis. The miRNA−mRNA, miRNA−lncRNA, and miRNA−circRNA pairs were estimated *via* the psRobot (Wu et al., 2012). Pairwise associations among the miRNA−mRNA, miRNA−lncRNA, and miRNA−circRNA relationships were assessed *via* the Spearman correlation coefficient (SCC) and paired expression profile data (He et al., 2020). The association axis was generated with the Cytoscape software (https://cytoscape.org).

### Real-time quantitative PCR based verification

Real-time qRT-PCR was conducted with BIO-RAD CFX Connect^™^ Optics Module (Bio-Rad, USA). Extracted RNA (1 μg) from sorghum was used to contract cDNA with the superscript first-strand synthesis system (PrimeScript^®^ RT Reagent Kit With gDNA Eraser, TaKaRa, Japan). Primer-BLAST in NCBI (https://www.ncbi.nlm.nih.gov/tools/primer-blast/) was used to design specific primers. *GAPDH* (circRNA, lncRNA, and mRNA) and *U6* (for miRNA) served as the endogenous controls. The employed primer sequences are summarized in **Supplemental Table S1**.

### Statistical analysis

Data analyses were performed *via* Student’s t test (two-tailed) using the SPSS v.20 (IBM Corp, Armonk, NY, USA) and GraphPad Prism 8.0 software (GraphPad Inc., La Jolla, CA, USA), and are presented as mean ± SEM. ^*^ *p* < 0.05 and ^**^ *p* < 0.01 indicated significance.

## Results

### Morpho-physiological and leaf ultrastructure variations within sorghum (“Jitian 3”) exposed to GA_3_ under salt stress

**Figure 1:**
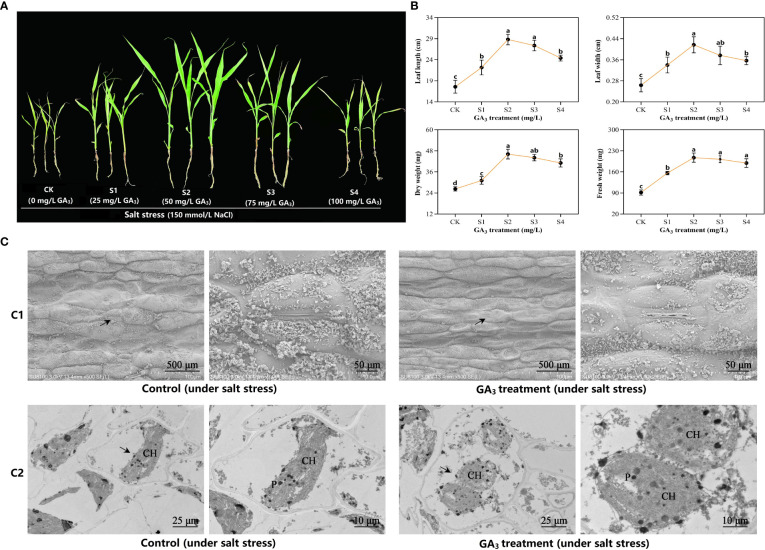
Sorghum phenotypic and leaf ultrastructure differences after exposure to exogenous gibberellic acid (GA_3_) under salt-stress. **(A)** Phenotypic observation. All sorghum plants were exposed to 150 mM NaCl to induce salt stress. CK refers to the control plants under salt stress. S1–S4 denotes the experimental leaves with 25 mg/L GA_3_, 50 mg/L GA_3_, 75 mg/L GA_3_, and 100 mg/L GA_3_ exposure, respectively, under salt stress. **(B)** Morphologic variations. Six sorghum plants were selected from the GA_3_-treated (n= 3) and control groups (n= 3) for morphologic evaluation, which included assessment of the plant leaf width, leaf length, as well as fresh and dry weights. All data are provided as mean ± SD, and Superscript distinct small letters represent a marked difference (*p*< 0.05), whereas, superscript same small letters represent no obvious difference (*p*> 0.05). **(C)** The 50 mg/L GA_3_-mediated regulation of sorghum leaf anatomical structure under salt stress. C1—epidermal structure and stomatal status; C2—cellular ultrastructure. The figure on the right is an enlarged vision of the black arrow-labeled area on the left. CH, chloroplast; P, plastoglobuli.

We examined the phenotypic variations of sorghum following exposure to different concentrations of GA_3_. GA_3_ application significantly promoted sorghum development at 50 mg/L GA_3_ treatment (S2) (**Figure 1A**), and enhanced the values for several characteristics, including leaf width, leaf length, as well as fresh and dry weights (**Figure 1B**). To further assess the 50 mg/L GA_3_-mediated effect on sorghum leaf ultrastructure, we examined the mesophyll cells and sorghum leaf chloroplasts *via* TEM. As depicted in **Figure 1C**, salt stress closed almost all sorghum leaf stomata in control plants. Interestingly, in the 50 mg/L GA_3_-treated plants, the stomatal aperture was considerably broader than in controls. Based on our TEM results, the mesophyll cells showed marked deformity under salt stress, and they were abnormally shaped. Moreover, relative to controls, the mesophyll cell ultrastructure in the 50 mg/L GA_3_–treated leaves and the chloroplasts and starch granules was intact.

### Screening and functional enrichment analysis of DE-mRNAs from the GA_3_ treated (GL_A) and control (GL_B) samples

**Figure 2:**
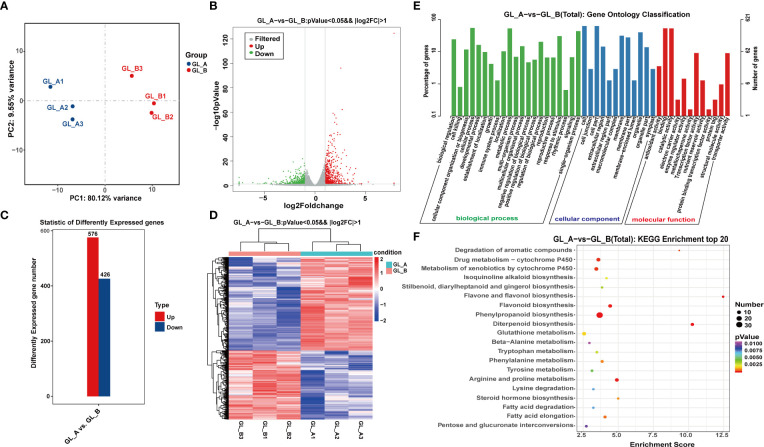
Screening and analysis of the GA_3_-treated Sorghum (“Jitian 3”) mRNA with under salt stress. **(A)** Principal Component Analysis (PCA) of GA_3_-treated (GL_A) and control (GL_B) samples; Blue dots represent GL_A samples, and red dots represent GL_B samples. **(B)** A volcano plot displaying the differentially expressed mRNAs (DE-mRNA) between the GL_A (n = 3) and GL_B groups (n = 3) based on RNA-seq analysis. Red dots represent upregulated DEGs; green dots represent downregulated DEGs; dotted line represents a screening threshold for DEGs; the x-axis values correspond to the log2 (fold change) value; the y-axis corresponds to the mean expression value of the −log10 (p-value) between the GA_3_-treated (GL_A) and control (GL_B) groups (fold change = GL_A/GL_B). **(C)** Statistical analysis of the DE-mRNAs in the GL_A and GL_B samples. **(D)** Hierarchical cluster analysis of DE-mRNAs among the six mRNA sequencing libraries; Red indicates upregulation, while blue indicates downregulation. GL_A1, GL_A2 and GL_A3 represent the three replications of GA_3_-treated samples; GL_B1, GL_B2 and GL_B3 represent the three replications of control samples. **(E)** gene ontology (GO) enrichment analysis; The x-axis shows the second level GO terms from the biological process (BP), cellular component (CC) and molecular function (MF) while the y-axis shows the number and percentage of gene enrichment. **(F)** Kyoto encyclopedia of genes and genome (KEGG) network enrichment analysis of DE-mRNAs. The x-axis showed an enrichment factor and the y-axis showed the pathway name; the point size represents the number of DE-mRNAs and the point color represents the p-value range.

Employing the Illumina HiSeq 2500 platform, we conducted the whole-transcriptome sequencing of six RNA libraries (GL_A1, GL_A2, GL_A3, GL_B1, GL_B2, and GL_B3). In all, we acquired 304.39 and 305.03 million raw reads and 299.65 and 300.26 million clean reads following filtration from the GL_A and GL_B libraries, respectively (**Supplementary Table S2**). Post quality control, principal component analysis (PCA) revealed good sample repeatability in each group (**Figure 2A**), which could be utilized in further analysis. In addition, |log2 (fold change) | > 1 and *p* ≤ 0.05 served as the standard cut-off for differentially expressed (DE) mRNAs (DE-mRNAs) screenings (**Figure 2B**). Overall, we identified 1002 DE-mRNAs, among which 576 were highly expressed (57.49%) and 426 were scarcely expressed (42.51%) from the GL_A group (**Supplementary Table S3**; **Figure 2C**). The DE-mRNAs expression profiles of both groups were visualized *via* a heat map. As illustrated in **Figure 2D**, the GL_A and GL_B DE-mRNAs were separately clustered. However, for each of these, the three replicates were clustered together. To explore the potential roles of these DE-mRNAs, we conducted Gene Ontology (GO) and Kyoto Encyclopedia of Genes and Genome (KEGG) enrichment analyses (**Figures 2E, F**). Most DE-mRNAs received annotations to GO terms “biological regulation”, “developmental process” and “response to stimulus” under biological process (BP); to “membrane”, “organelle” and “extracellular region” under cellular component (CC); and to “binding”, “antioxidant activity” and “transcription factor activity” under molecular function (MF) (**Figure 2E**). Based on the KEGG analysis, the DE-mRNAs enrichments were in 97 networks, including 41 strongly enriched KEGG axes (*p* ≤ 0.05) (**Supplementary Table S4**; **Figure 2F**). Among them, the “phenylpropanoid biosynthesis” (ko00940) pathway was markedly enriched in the GL_A and GL_B samples, and this pathway strongly modulates sorghum development and response to salt stress.

### Identification and FEA of DE-lncRNAs in the GA_3_-treated (GL_A) and control (GL_B) samples

**Figure 3:**
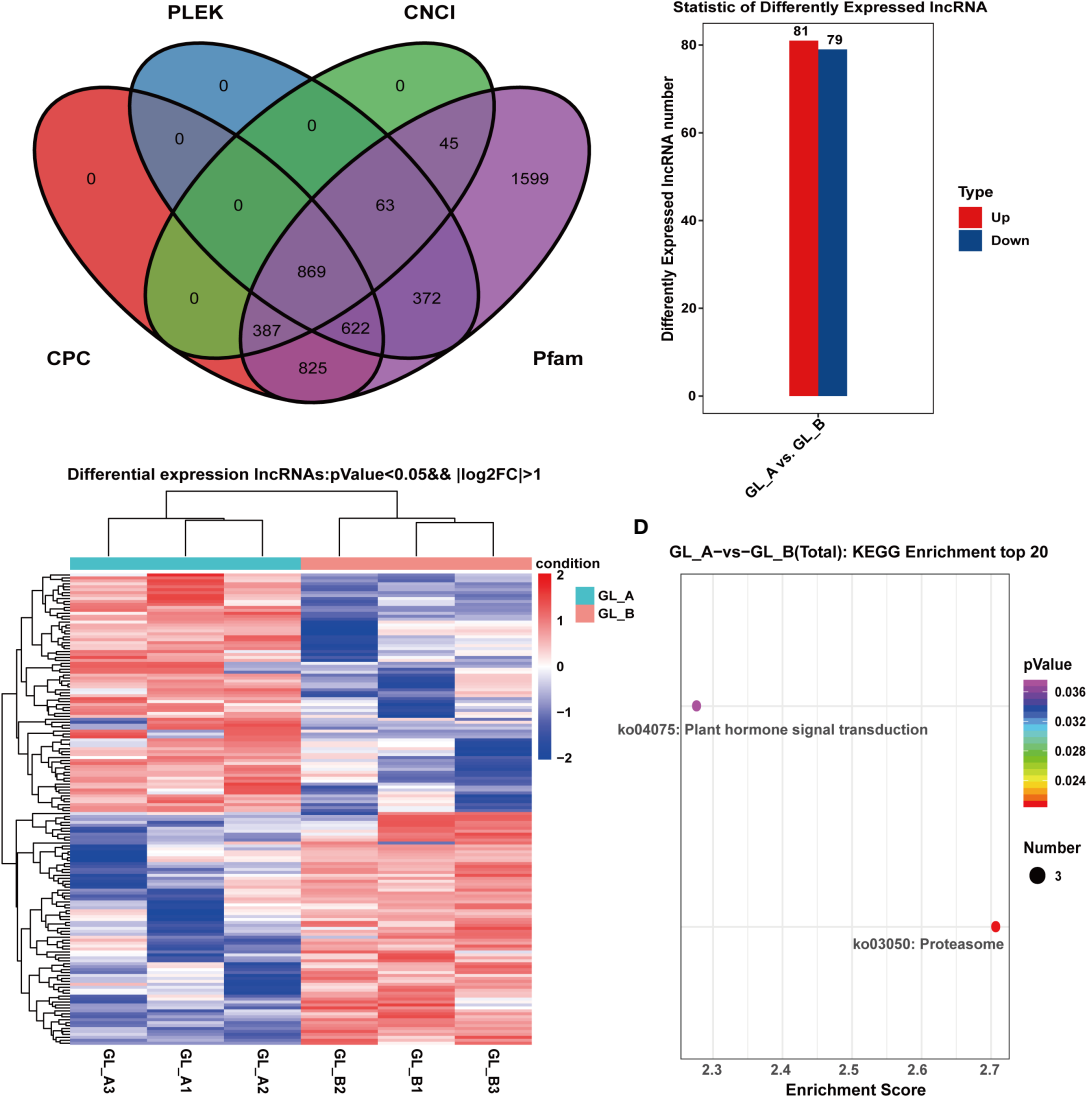
Screening and analysis of long-coding RNAs (lncRNAs) in the GA_3_-treated Sorghum (“Jitian 3”) with under salt stress. **(A)** Coding potential analysis of lncRNAs based on four computational approaches (CNCI, CPC, PFAM, and PhyloCSF) by venny software (https://bioinfogp.cnb.csic.es/tools/venny/index.html). **(B)** Statistical analysis of the differentially expressed lncRNAs (DE-lncRNAs) in the GL_A and GL_B samples; Red dots represent upregulated DE-lncRNAs; blue dots represent downregulated DE-lncRNAs. **(C)** Heatmap analysis of DE-lncRNAs among the six lncRNA sequencing libraries; Red indicates upregulation, while blue indicates downregulation. **(D)** KEGG network enrichment analysis for all DE-lncRNA targets. Scatter plot of selected signifcantly targets pathway terms for the 160 diferentially expressed lncRNAs. Counts represent the numbers of DE-lncRNA targets.

Along with the mRNAs, we identified 869 lncRNAs employing four methods of CNCI (Sun et al., 2013), CPC (Kong et al., 2007), Pfam (Mistry et al., 2021) and PLEK (Li et al., 2014) for subsequent analyses (**Figure 3A**). In all, 160 DE-lncRNAs were identified in the GL_A and GL_B samples using the following parameters: | log2 (fold change) | > 1 and *p* ≤ 0.05. Out of the 160 DE-lncRNAs, 81 were highly expressed (50.62%), and 79 were scarcely expressed (49.38%) in the GL_A group (**Figure 3B**; **Supplementary Table S5**). **Figure 3C** illustrates the expression patterns of the identified DE-lncRNAs. To elucidate the physiological role of DE-lncRNAs, we performed KEGG enrichment analyses of the DE-lncRNA-targeted DE genes (**Supplementary Table S6**). As shown in **Figure 3D**, the DE-lncRNAs target genes were strongly enriched in two essential networks, namely, proteasome (ko03050) and plant hormone axes (ko04075).

**Figure 4:**
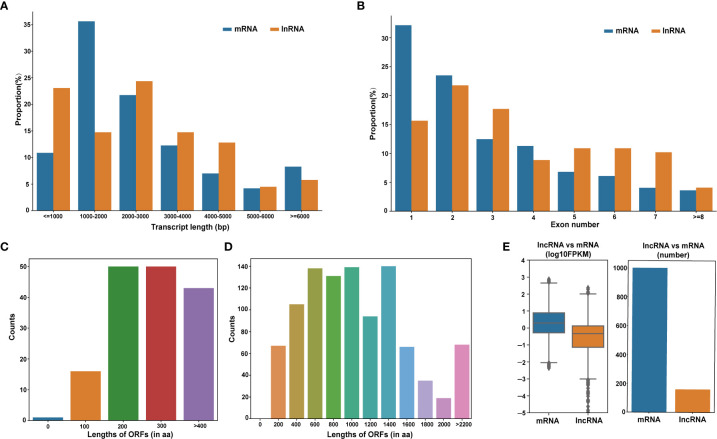
Evaluation of the DE-lncRNA and DE-mRNA structural and expression profiles. **(A)** DE-lncRNAs and DE-mRNAs transcript length distributions. **(B)** Structural comparison between lncRNAs and mRNAs in terms of exon number. **(C, D)** Open reading frames (ORF) length distribution of DE-lncRNAs and DE-mRNAs. **(E)** DE-lncRNA and DE-mRNA expression levels. DE-lncRNAs, differentially expressed long-coding RNAs; DE-mRNAs, differentially expressed mRNAs.

LncRNAs cis-regulate nearby genes in order to transcriptionally or post-transcriptionally modulate gene expression (Ponjavic et al., 2009). Based on the genomic assessments of DE-lncRNAs and DE-mRNAs, long (< 5000 bp) DE-mRNAs were more prevalent, relative to the long DE-lncRNAs (**Figure 4A**). The mean DE-mRNAs open reading frame (ORF) was longer than the mean DE-lncRNAs ORF. The DE-lncRNAs ORFs were estimated to be between 200−300 aa long (**Figure 4C**), while most DE-mRNAs ORFs were between 400−1400 aa long (**Figure 4D**). Moreover, the DE-mRNAs on average possessed fewer exons (1–2), compared to the DE-lncRNAs (**Figure 4B**), and the DE-mRNAs expressions were elevated, compared to the DE-lncRNAs (**Figure 4E**).

### Screening analysis of DE-circRNAs in the GA_3-_treated (GL_A) and control (GL_B) samples

**Figure 5:**
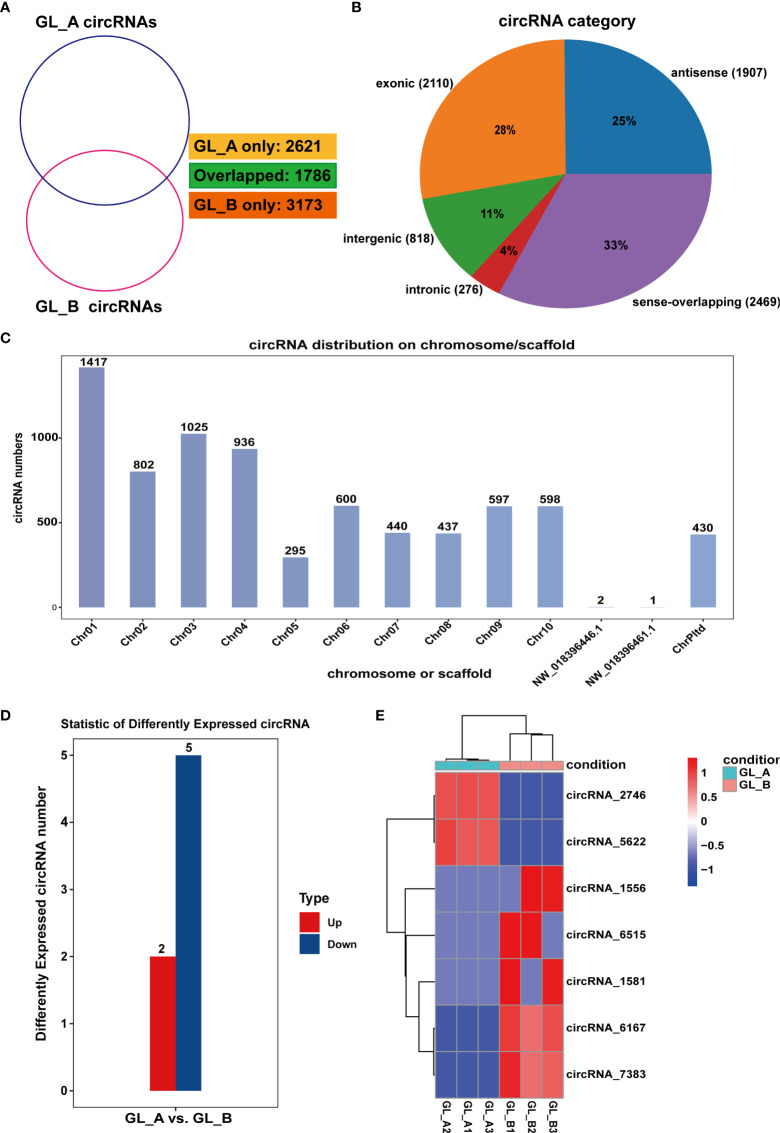
Screening and analysis of circular RNAs (circRNAs) in the GA_3_-treated Sorghum (“Jitian 3”) under salt stress. **(A)** Venn diagram depicting the number of circRNAs in the GA_3_-treated (GL_A) and control (GL_B) samples. **(B)** circRNAs category analysis. **(C)** Distribution of circRNAs on the *Sorghum bicolor* chromosome. **(D)** Statistic analysis of the number of differentially expressed circRNAs (DE-circRNAs) from the GL_A and GL_B samples. **(E)** Heatmap analysis of DE-circRNAs among the six circRNA sequencing libraries; Red indicates upregulation in GL_A group, while blue indicates downregulation in GL_A group.

Overall, 7580 circRNAs were screened in the GL_A and GL_B samples, among which 2621 and 3173 were unique to the GL_A and GL_B samples, respectively (**Figure 5A**). All 7580 circRNAs were classified into five categories, including exonic (2110), antisense (1907), intergenic (818), intronic (276) and sense-overlapping (2469) (**Figure 5B**). The circRNAs were extensively distributed on different chromosomes, except for NW_018396446.1 and NW_018396461.1 (**Figure 5C**). Following identification, 7 DE-circRNAs were acquired (|log2 (fold change)| > 1 and *p* ≤ 0.05), which included 2 highly and 5 scarcely expressed DE-circRNAs in the GL_A group (**Supplementary Table S7**; **Figure 5D**). A heat map of the DE-circRNAs was generated to display the DE-circRNA expression profiles in the individually treated samples (**Figure 5E**).

### Screening and FEA of DE-miRNAs in the GA_3_-treated (GL_A) and control (GL_B) samples

**Figure 6:**
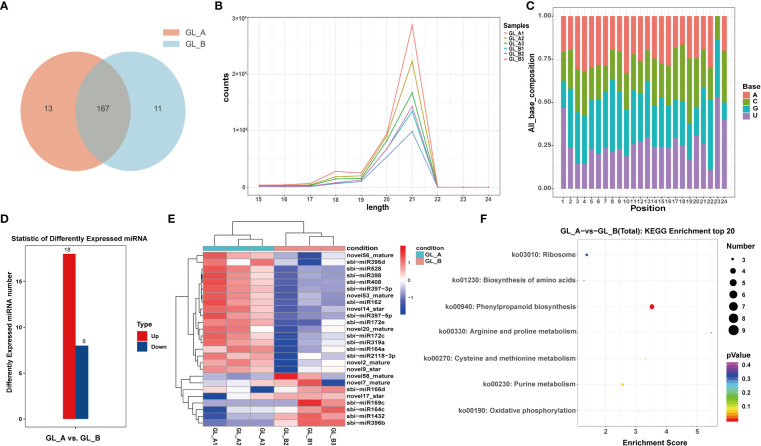
Screening and analysis of microRNAs (miRNAs) in the GA_3_-treated sorghum (“Jitian 3”) under salt stress. **(A)** Venn diagram illustrating the number of miRNAs in GA_3_-treated (GL_A) and control (GL_B) samples. **(B, C)** Sequence length distribution of small RNAs isolated from the six sorghum leaf libraries **(A)** and the nucleotide compositions of the mature miRNAs **(B)**; GL_A1, GL_A2 and GL_A3 refer to the three GA_3_-treated sample replicates; GL_B1, GL_B2 and GL_B3 refer to the three control sample replicates. **(D)** Statistic analysis of the number of differentially expressed miRNAs (DE-miRNAs) in the GL_A and GL_B groups; Red represents upregulation in GL_A group, while blue represents downregulation in GL_A group. **(E)** Heatmap analysis of DE-miRNAs among the six miRNA sequencing libraries. **(F)** KEGG network enrichment analysis of all DE-miRNAs targets.

To conduct an extensive analysis of the miRNA repertoire associated with the GA_3_-mediated regulation of salt stress, the GL_A and GL_B libraries were generated and subsequently sequenced. Upon filtration, 27,749,206 and 26,415,849 unique reads were retrieved from the GL_A and GL_B libraries, respectively. Overall, we screened 191 miRNAs, among which 13 and 11 were specific to the GL_A and GL_B samples, respectively (**Figure 6A**). The 21-nt reads were commonly found among all six libraries, followed by the 20-nt lengths (**Figure 6B**). This results suggested that more post-transcriptional modifcations may exist in Sorghum (“Jitian 3”) because the 21-nt sRNAs form the majority of small interfering RNAs (Xie et al., 2015). Based on our miRNA bias analysis, mature miRNAs did not typically begin with varying bases (A, C, G or U) (**Figure 6C**). In all, 26 DE-miRNAs with (|log2 (fold change)| > 1 and *p* ≤ 0.05) were screened, among which 18 were highly (69.23%) and 8 were scarcely expressed (30.77%) (**Supplementary Table S8**; **Figure 6D**). Moreover, like the DE-mRNAs and DE-lncRNAs, the DE-miRNAs in the GL_A and GL_B samples were independently clustered, and individual cases of three replicates were clustered together (**Figure 6E**). Furthermore, we estimated the target mRNAs of the identified DE-miRNAs using KEGG analyses. The DE-miRNAs target genes were enriched in 7 networks (**Supplementary Table S9**; **Figure 6F**). Among them, two significant pathways related to sorghum growth and salt stress response were the “phenylpropanoid biosynthesis (ko00940)” and “arginine and proline metabolism” axes (*p* < 0.05).

### ceRNA network analysis

**Figure 7:**
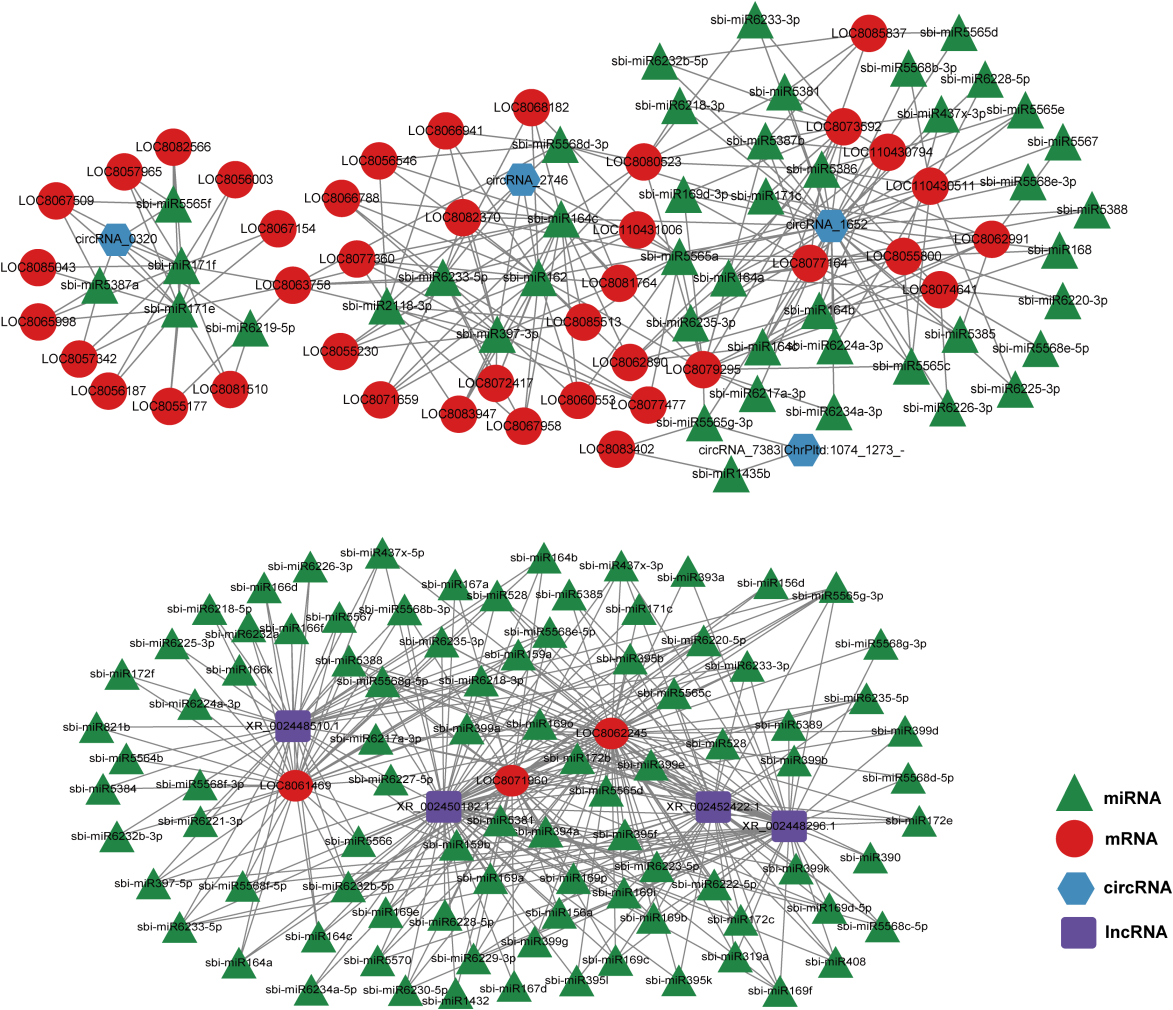
Competitive endogenous RNA (ceRNA) network generated using the differentially expressed (DE)-mRNAs, DE-lncRNAs, DE-circRNAs, and DE-miRNAs in sorghum (“Jitian 3”) between GL_A and GL_B groups. Red, blue, and green represent lncRNA, miRNAs, and mRNAs, respectively. ceRNA network was conducted by Cytoscape software (https://cytoscape.org/).

LncRNAs and circRNAs typically bind to the miRNA response elements in miRNAs, as part of the ceRNA axis (Salmena et al., 2011). To identify the overall modulatory axis of the protein-coding RNAs and ncRNAs associated with the GA_3_-mediated regulation of salt stress, ceRNA networks were generated with DE-mRNAs, DE-miRNAs, DE-lncRNAs, and DE-circRNAs, according to the ceRNA theory using the cytoscape software (https://cytoscape.org) (**Figure 7**). Based on the ceRNA network characteristics, we revealed three circRNAs (circRNA_2746, circRNA_6515, and circRNA_5622) and four lncRNAs (XR_002450182.1, XR_002452422.1, XR_002448510.1, and XR_002448296.1) in the core of the network, which may serve critical roles in modulating sorghum development.

### RNA-seq result confirmation using qRT-PCR

**Figure 8:**
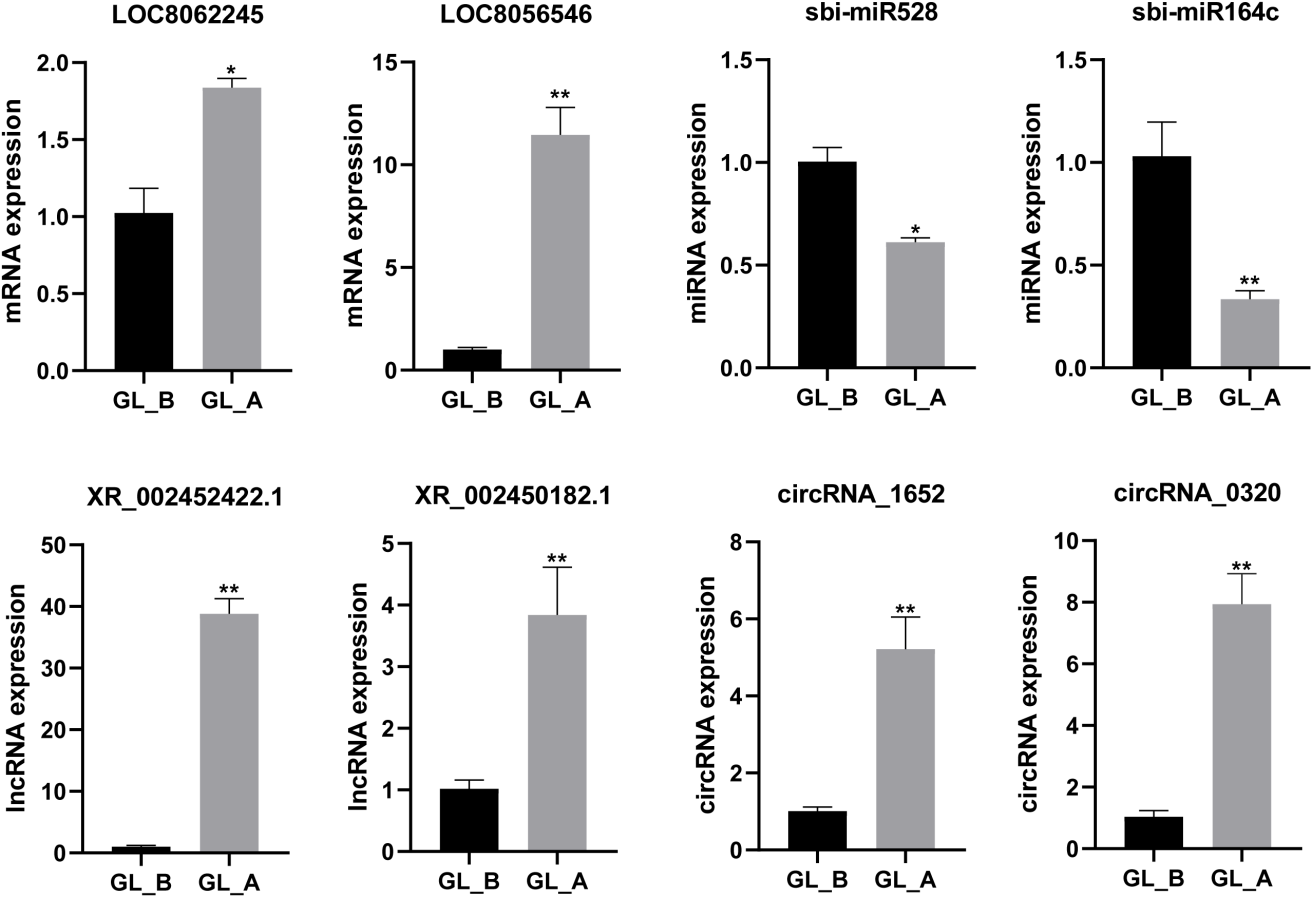
Quantitative real-time PCR (qRT-PCR)-mediated confirmation of select differentially expressed (DE)-mRNAs, DE-miRNAs, DE-lncRNAs, and DE-circRNAs in sorghum (“Jitian 3”) between GL_A and GL_B groups. All data are provided as mean ± SEM, n = 3, *p < 0.05, **p < 0.01.

To further confirm the RNA-Seq results, we arbitrarily chose two DE-mRNAs (LOC8056546, LOC8062245), two DE-miRNAs (sbi-miR164c, sbi-miR528), two DE-lncRNAs (XR_002450182.1, XR_002452422.1), and two DE-circRNAs (circRNA_1652, circRNA_0320) for qRT-PCR analysis. Our findings revealed that the expression profiles were comparable to the whole-transcriptome data, indicating the dependability of the RNA sequencing results (**Figure 8**).

## Discussion

Plants are highly vulnerable to abiotic stressors in early development. Salt stress is a key factor limiting sorghum germination under saline conditions. Previous studies concluded that low concentrations (< 50 mM) of NaCl promotes germination, while high concentrations (> 100 mM) of NaCl significantly inhibits germination (Rubio et al., 2020). Exogenous plant growth phytohormone administration was shown to be highly efficacious in alleviating the negative impacts of salinity. Among them, gibberellins are established promoters of plant growth under saline stress (Camara et al., 2018; Muniandi et al., 2018). Although salinity stress induces multiple adverse reactions in plants, gibberellins can remove the harmful substances brought on by salt stress, and maintain intercellular stability by modulating photosynthesis, the antioxidant system, osmotic substances, and ion balance (Jiao et al., 2019). Herein, we examined the influence of various GA_3_ concentrations on the morpho-physiological and leaf ultrastructure variations of sorghum (“Jitian 3”) under high salinity conditions. We next confirmed the optimal GA_3_ concentration (50 mg/L) required for minimizing most salt stress. In contrast, exogenous gibberellin administration promotes the essential protein synthesis needed for sorghum growth and endohydrolase activity, which has positive effects on augmenting plant height and resisting salt stress in sorghum (Kim et al., 2009). It is well known that plants have a complex response to salt stress. Under salt stress, plants first respond at the genetic level using transcriptional regulation, then they synthesize RNA coding for proteins associated with salt stress, and lastly, they utilize fine control of metabolite biosynthesis, which regulates plant metabolism and osmotic balance (Hasanuzzaman and Fujita, 2022).

Over the past decade, high-throughput approaches have enriched the identification of key elements in plant stress tolerance (Hernández et al., 2017). A better comprehension of the tolerance molecular mechanism can advance the development of salt-resistant sorghum lines using genetic engineering. Furthermore, the extensive screening and analysis of ceRNA axes associated with GA_3_ modulation remains incomplete. Prior reports revealed that lncRNAs and circRNAs (as ceRNAs) modulate one another *via* association with shared miRNA response elements (Ala et al., 2013). Hence, the ceRNAs modulatory axis, generated by lncRNAs, circRNAs, miRNAs, and mRNAs associating with miRNA response elements is critical to the post-transcriptional gene modulation in numerous biological processes. In this study, we employed a comparative whole-transcriptome analysis to reveal 1002 DE-mRNAs, 81 DE-lncRNAs, 26 DE-miRNAs, and 7 DE-circRNAs in GA_3_-treated samples, compared to controls. We generated the first ever GA_3_-associated ceRNA-miRNA-target gene modulatory axis to offer a foundation for additional investigation on the underlying mechanism behind salt stress regulation. We also conducted KEGG analyses to examine the likely roles of DE-mRNAs, DE-lncRNAs, DE-circRNAs, and DE-miRNAs targets. Following GA_3_ exposure, the enriched networks including numerous genes correlated with the phenylpropanoid biosynthesis and plant hormone axes. The phenylpropanoid pathway serves an essential function in plant development as well as their response to environmental stress (Sharma et al., 2019). Plant hormones mediate salinity signals to modulate plant growth adaptation, which play an essential role in regulating salt responses (Yu et al., 2020). Associations among mRNAs, miRNAs, lncRNAs, and circRNAs modulate gene expression, and thus, the competitive endogenous RNA (ceRNA) hypothesis was put forth (Salmena et al., 2011). The ceRNA axis was generated and examined in numerous plants, demonstrating crucial functions in plant growth (Fan et al., 2018; Xu et al., 2016). Nevertheless, the ceRNAs modulatory network belonging to the vernalization axis remains undetermined. Therefore, a closer examination of the associations between mRNAs, miRNAs, lncRNAs, and circRNAs in sorghum development and stress response is both urgent and necessary. In this study, we conducted the ceRNA network and screened three circRNAs (circRNA_2746, circRNA_6515, circRNA_5622), four lncRNAs (XR_002450182.1, XR_002452422.1, XR_002448510.1, XR_002448296.1), four genes (LOC8056546, LOC8062245, LOC8061469, LOC8071960) as valuable candidates for regulating the GA_3_-mediated alleviation of salt stress in sorghum. However, limited investigations assessed the function of the aforementioned lncRNAs and circRAs in sorghum leaf development. Additional investigations are necessary to fully elucidate the activities of these GA_3_ regulation-associated ceRNAs, ncRNAs, and target genes in modulating sorghum salt response.

## Conclusion

In conclusion, a whole-transcriptome sequencing was performed using GA_3_-treated and control sorghum (“Jitian 3”) leaves under salt stress, and a total of 1002 DE-mRNAs, 81 DE-lncRNAs, 26 DE-miRNAs, and 7 DE-circRNAs were identified. Functional analyses revealed that DE-mRNAs and DE-ncRNAs target mRNAs were primarily enriched in the phenylpropanoid biosynthesis, plant hormone proteasome, as well as arginine and proline metabolism axes. Moreover, we constructed a circRNA/lncRNA−miRNA–target gene regulatory ceRNA network in sorghum, and identified several ncRNA (circRNA_2746, circRNA_6515, circRNA_5622, XR_002450182.1, XR_002452422.1, XR_002448510.1, XR_002448296.1) and mRNAs (LOC8056546, LOC8062245, LOC8061469, LOC8071960) that contribute to the GA_3_-mediated alleviation of salt stress in sorghum (“Jitian 3”). This evidence highlights the importance of mRNAs and ncRNAs in the molecular regulation of salt stress response in sorghum.

## Data availability statement

The original contributions presented in the study are publicly available. This data can be found here: NCBI, PRJNA878791 and PRJNA858876.

## Author contributions

YW and GZ planned and designed the ex*p*eriments. YW and JL performed the experiments. YW analyzed the data and wrote the manuscript. All authors contributed to the article and approved the submitted version.

## Funding

This work was supported by the China National Key R&D Program (2022YFE0113400), the National Natural Science Funds (32102411), the Natural Science Foundation of Jiangsu Province of China (BK20200924), the Natural Science Foundation of Jiangsu Higher Education Institutions of China (20KJB210005), Jiangsu Association for Science and Technology young Scientific and technological Talents Project—supported by Yanqing Wu.

## Acknowledgments

We thank OE Biotech Co., Ltd (Shanghai, China) for RNA-seq and Zhou Xuan for assistance with data upload.

## Conflict of interest

The authors declare that the research was conducted in the absence of any commercial or financial relationships that could be construed as a potential conflict of interest.

## Publisher’s note

All claims expressed in this article are solely those of the authors and do not necessarily represent those of their affiliated organizations, or those of the publisher, the editors and the reviewers. Any product that may be evaluated in this article, or claim that may be made by its manufacturer, is not guaranteed or endorsed by the publisher.

## Supplementary material

Supplementary Table 1 Real-time PCR primers and sequences.

Supplementary Table 2 Summary of sequencing data from whole-transcriptome sequencing.

Supplementary Table 3 Summary of differentially expressed (DE)-mRNAs identified in the GA_3_-exposed (GL_A) and control (GL_B) sorghum (“Jitian 3”).

Supplementary Table 4 KEGG network enrichment analysis of the differentially expressed (DE) mRNAs in sorghum (“Jitian 3”).

Supplementary Table 5 A list of differentially expressed (DE)-lncRNAs in the GA_3_-exposed (GL_A) and control (GL_B) sorghum (“Jitian 3”).

Supplementary Table 6 KEGG network enrichment analysis of the differentially expressed (DE)-lncRNAs target genes in sorghum (“Jitian 3”).

Supplementary Table 7 A list of differentially expressed (DE)-circRNAs in the GA_3_-exposed (GL_A) and control (GL_B) sorghum (“Jitian 3”).

Supplementary Table 8 A list of differentially expressed (DE)-miRNAs in the GA_3_-exposed (GL_A) and control (GL_B) sorghum (“Jitian 3”).

Supplementary Table 9 KEGG network enrichment analysis of the differentially expressed (DE)-miRNAs target genes in sorghum (“Jitian 3”).
